# Enhanced Antitumor Activity of Lidocaine Nanoparticles Encapsulated by a Self-Assembling Peptide

**DOI:** 10.3389/fphar.2022.770892

**Published:** 2022-04-21

**Authors:** Yang Yang, Jiaxiao Sun, Fei Peng, Haibei Liu, Guoyan Zhao, Junjie Chen, Wensheng Zhang, Feng Qiu

**Affiliations:** ^1^ Department of Anesthesiology, West China Hospital, Sichuan University, Chengdu, China; ^2^ Laboratory of Anesthesia and Critical Care Medicine, National-Local Joint Engineering Research Centre of Translational Medicine of Anesthesiology, West China Hospital, Sichuan University, Chengdu, China; ^3^ Department of Burn and Plastic Surgery, West China Hospital, Sichuan University, Chengdu, China

**Keywords:** lidocaine, antitumor activity, self-assembling peptide, local anesthetics, nanoparticles, tumor recurrence

## Abstract

Although local anesthetics (LAs) such as lidocaine have been traditionally used for pain relief, their antitumor activity has attracted more and more attentions in recent years. However, since nearly all LAs used in clinic are in their hydrochloride forms with small molecular weight and high water-solubility, their fast absorption and clearance greatly limit their antitumor activity *in vivo*. To better exploit the antitumor activity of LAs, lidocaine nanoparticles (LNPs) are prepared by using a self-assembling peptide to encapsulate the hydrophobic base form of lidocaine. In cultured A375 human melanoma cells, the LNPs show much higher cellular uptake level than the clinic formulation of lidocaine hydrochloride, which leads to enhanced efficacy in inhibiting the proliferation, migration and invasion of the cells, as well as in inducing cell apoptosis. Compared with lidocaine hydrochloride, LNPs can also significantly slow down the release rate of lidocaine. In nude mice, LNPs can effectively inhibit the development of solid tumors from seeded A375 cells and prevent the recurrence of tumors after surgical excision. These results indicate that by using self-assembling peptide to fabricate nanoparticle formulations of local anesthetics, their antitumor activity can be significantly enhanced, suggesting a potential postoperative treatment to prevent tumor recurrence after surgical excision.

## 1 Introduction

Local anesthetics (LAs) are a group of small molecular drugs widely used for anesthesia and analgesia in clinic practice. Compared with traditional pain-relieving opioids, LAs are relatively safe and non-addictive, so that more and more frequently have they become the choice of physicians for the treatment of perioperative and postoperative pain (Farag et al., 2013; Guay et al., 2016; Svirskis et al., 2020).

Surgical excision of solid tumor for curing cancer is one of the most important and common kinds of operations routinely carried out in clinical practice. With the extensive involvement of LAs in cancer-related surgeries, more and more clinical evidences have implied that the use of LAs during or after the surgery was usually associated with improved outcome of patients (Call et al., 2015; Royds et al., 2016; Zhang et al., 2021). It is generally accepted that potential benefits of LAs include relieving the pain, reducing the stress caused by surgery, blunting the inflammatory response, thus improving the overall rehabilitation quality of the patient (Yang et al., 2017; Müller et al., 2021). Nevertheless, some studies have also suggested that LAs, if in direct contact with tumor cells, may also kill them directly (Liu et al., 2020).

Following these findings, more and more studies have been carried out on the direct antitumor activity of LAs. To date, many conventional LAs including lidocaine, bupivacaine, ropivacaine, and mepivacaine have shown antitumor activity against different tumor cells including lung cancer, breast cancer, colon-rectal cancer, melanoma, et al. (Bundscherer et al., 2015; Chamaraux-Tran et al., 2018; Li et al., 2018; Chen et al., 2019; Siekmann et al., 2019). Extensive mechanism studies have indicated that LAs may exert their antitumor activity through various pathways such as ion channel blocking (Baptista-Hon et al., 2014; Fraser et al., 2014), microRNA regulation (Qu et al., 2018; Sui et al., 2019; Xia et al., 2019), inflammation inhibiting (Piegeler et al., 2012; Wall et al., 2019), and so on.

However, on the contrary to the extensively reported antitumor activity of LAs on cultured tumor cell lines *in vitro*, only a few animal studies have been reported to show their antitumor activity *in vivo*. There seems to be a huge gap between the confirmed cytotoxicity of LAs against tumor cells and their actual antitumor activity *in vivo*. One possible reason is that all LAs in their clinical formulations are highly water-soluble small molecules so that they tend be diffused, absorbed, and cleared out very quickly in body before they can accumulate a high enough antitumor effect. Interestingly, in those studies reporting antitumor activity of LAs *in vivo*, the drugs were all encapsulated in nanoparticles based on carrier materials (Gao et al., 2018; Zhang et al., 2020), suggesting that nanoparticle formulations might be a promising strategy to improve the antitumor activity of LAs. However, in these studies LAs were combined with other treatments such as nutrient deprivation and classic antitumor drugs, and LAs seemed to only play a supplementary role. The antitumor activity of LAs alone, if encapsulated as nanoparticles, has not been evaluated yet.

As an emerging category of biomaterials with excellent biocompatibility and controllability, self-assembling peptide nanomaterials have shown great potential as advanced drug carriers (Qiu et al., 2018; Peng et al., 2020). In our previous study, a self-assembling peptide GQY with the sequence of GQQQQQY was designed to form nanoparticles with very high drug-loading capacity (Liu et al., 2021). This biocompatible nanomaterial can readily encapsulate hydrophobic drugs into nanoparticles, providing a simple strategy to fabricate drug nanoparticles. Although LAs currently used in clinic practice are their soluble hydrochlorides, their base forms are hydrophobic molecules with poor water solubility, making it possible to get LA nanoparticles using GQY as the carrier material. To test the feasibility of this strategy, lidocaine as one of the most widely used LAs with well-defined antitumor activity was used as a model drug. In this study, we show how lidocaine base (LB) can be encapsulated with GQY and form lidocaine nanoparticles (LNPs), which show enhanced antitumor activity against human melanoma cell line A375 *in vitro* and effectively inhibit the development and recurrence of solid tumors *in vivo*.

## 2 Materials and Methods

### 2.1 Sample Preparation

#### 2.1.1 Peptide and Reagents

GQY peptide with purity of 98% was synthesized by Bootech BioScience and Technology Co., Ltd. (Shanghai, China) and provided as lyophilized powder. Lidocaine hydrochloride (LH) (purity ≥99%) and LB (purity ≥99%) were purchased from Aladdin Biochemical Technology Co. Ltd (Shanghai, China). Thioflavin T (ThT) was purchased from Sigma-Aldrich Co. (St Louis, MO, United States).

#### 2.1.2 Preparation of LNP Suspension and LH Solution

GQY was dissolved in sterile 10 mM phosphate buffer with a pH value of 7.8 to obtain a working solution with a peptide concentration of 5 mM. LB powder was added into the GQY solution at a theoretical lidocaine concentration of 100 mM, then the mixture was vigorously stirred at room temperature for 24 h to obtain a stable milky suspension. Transparent LH solution with a concentration of 100 mM was obtained by directly dissolving LH powder in sterile Milli-Q water.

### 2.2 Characterization of Nanostructures

#### 2.2.1 Transmission Electron Microscope

LNP suspension or GQY solution was diluted ten-fold by Milli-Q water, and each 10 µL of diluted sample was set on the surface of a 400-mesh copper grid [Zhongjingkeyi (Beijing) Film Technology Co., Ltd]. After 2 min of incubation to deposit the sample, a piece of filter paper was used to blot the excess liquid. After that 10 µL of phosphotungstic acid (2%) was dropped onto the grid to stain the sample for 2 min. Finally, the excess staining solution was blotted with filter paper and the grid was air-dried. The grids were then observed and images were collected with a Tecnai G2 F20 transmission electron microscope (FEI, United States).

#### 2.2.2 Atomic Force Microscope

For AFM observation, each 10 µL of LNP or GQY sample diluted in the above section was spread on a freshly cleaved mica surface. After 2 min of incubation to deposit the sample, excess liquid was pipetted away and the mica surface was gently rinsed with 1 mL of Milli-Q water. After that the mica surface was air-dried and scanned with AFM (SPM-9700HT, Shimadzu, Japan) operated in tapping model.

#### 2.2.3 Dynamic Light Scattering

The size distribution and zeta potential of LNP and GQY nanoparticles were measured by DLS using a Zetasizer Nano ZS90 (Malvern Panalytical, Malvern, United Kingdom). Briefly, LNP suspension or GQY solution was diluted 10-fold with Milli-Q water and added into a disposable size cuvette or a potential cell for measurement. The size distribution plot and zeta potential plot were collected 3–5 times to make sure similar results were obtained.

#### 2.2.4 ThT-Binding Assay

ThT stock solution (1 mM) was prepared by dissolving ThT powder in Milli-Q water and passing through a 0.22 µm filter. To measure the ThT-binding fluorescence, 5 µL of ThT stock solution was mixed with 495 µL of LH, GQY or LNP, and the mixture was incubated at room temperature for 5 min. Fluorescent spectra between 460–600 nm were measured by a Fluorolog spectrofluorometer (Horiba scientific Inc., United States) with an excitation wavelength of 450 nm.

#### 2.2.5 Fourier Transform Infrared Spectrometer

For FTIR assay, dry powder of LB, LH, and GQY was used as received. LNP powder was obtained by lyophilization of the suspension. The physical mixture of LB and GQY (LB + GQY) was also used as a comparison. Each sample was mixed with KBr powder and compressed into a translucent thin film, and the FTIR spectra were collected with an IRTracer-100 spectrometer (Shimadzu, Japan).

### 2.3 Cell Experiments

#### 2.3.1 Cellular Uptake

A375 cells were purchased from Cell Center of Chinese Academy of Sciences (Shanghai, China) and conventionally maintained in DMEM containing 10% FBS (Thermo Fisher Scientific, MA, United States) and 1% penicillin/streptomycin (Invitrogen, CA, United States). To test the ability of GQY nanoparticles to deliver encapsulated drugs into cultured cells, insoluble doxorubicin (DOX) was prepared as previously described (Peng et al., 2021). Following the protocol described in Section 2.1.2, DOX was encapsulated in GQY to obtain a GQY-DOX nanoparticle stock suspension with a DOX concentration of 200 μg/mL. A375 cells suspended in 2 mL of medium were seeded into a glass-bottomed 35-mm dish (Nest Biotechnology, Wuxi, China) at a density of 2.5×10^4^ cells/mL and cultured overnight. Then fresh medium containing 10 μg/mL of GQY-DOX was added to the cells, which were kept in incubator for 4 h. The cells were then rinsed three times with PBS and stained with 20 μg/mL Hoechst (Sigma-Aldrich, St Louis, MO) for 20 min, following which the cells were rinsed three times with PBS and 5 μM ThT was used to stain the cells for another 5 min. After another three times of rinse with PBS, cells were imaged using an IXplore SpinSR confocal microscope (Olympus, Tokyo, Japan).

To compare the cellular uptake level of LH and LNP, A375 cells were seeded in 12-well plates at an initial density of 2.5×10^5^ cells/well and incubated overnight. Then the medium was replaced by fresh medium containing 6 mM of lidocaine as LH or in LNP and the cells were cultured for 4 h. Then the medium was removed and the cells were gently washed by PBS for 3 times to remove drug not absorbed by the cells. Three rounds of freeze-thaw cycle were then applied to induce cell lysis, and the amount of lidocaine in each well was determined by HPLC method.

#### 2.3.2 Cytotoxicity Assay

A375 cells were seeded into a 96-well plate at an initial density of 1×10^5^ cells/well and incubated overnight. To show the cytotoxicity of LH under short-time incubation, medium containing 1, 5 mM or 10 mM of LH was incubated with the cells for 4 h and then replaced by fresh medium without LH, and the cytotoxicity assay was carried out after another 20 h. Alternatively, medium containing 1, 5 mM or 10 mM of LH was incubated with the cells for 24 h until cytotoxicity assay was carried out. To compare the cytotoxicity of LH and LNP, medium containing 1–10 mM lidocaine as LH or in LNP was prepared by diluted the stock solution/suspension into fresh medium. Drug-containing medium was incubated with A375 cells for 4 h, after which the medium in each well was replaced by fresh medium without drug and the cells were cultured for another 20 h until cytotoxicity assay. After a total of 24 h of incubation, cell viability in each experiment was tested using an Enhanced Cell Counting Kit-8 (Saint-Bio, Shanghai, China) following the manufacture’s instruction. In all experiments, cells always incubated with drug-free medium were used as the control group, and medium without cells seeded was used as the blank. The optical density (OD) values were detected at 490 nm by an Eon microplate spectrophotometer (BioTek Instruments Inc., United States). Cell viability was calculated as followed:

$$\mathrm{Cell}\;\mathrm{viability}\;\left(\%\right)=\frac{\;\left(OD_{\mathrm{test}}\;–\;OD_{blank}\right)}{\;\left(OD_{control}\;–\;OD_{blank}\right)}\times 100\%$$

#### 2.3.3 Cell Migration

Migration of A375 cells under different treatments was assessed using a wound-healing assay as described previously (Li et al., 2018). The cells were cultured in 12-well plates and allowed to grow until reaching 95% confluency. The cell monolayer was scratched using a 200-µL pipet tip to create a “wound.” Then the cells were cultured in serum-free medium containing 6 mM of lidocaine as LH or in LNP for 4 h, after which the medium was replaced by serum-free medium without drug and the cells were culture until 24 h. Cells always cultured in serum-free medium containing no drug were used as control. Images of the wounds were taken at 0 and 24 h. The migration distance was analyzed with ImageJ.

#### 2.3.4 Cell Invasion

A375 cells were seeded in 6-well plate at a density of 6×10^5^ cells/well and cultured overnight. Then the medium was replaced by serum-free medium containing 6 mM lidocaine as LH or in LNP. Serum-free medium without drug was used as control. After 4 h of incubation, the cells in each well were washed with PBS and collected by trypsinization. Treated cells were suspended in serum-free medium and seeded into the Matrigel-coated upper chambers of transwells at a density of 2×10^4^ cells/well. The lower chamber of each well was filled with medium containing 10% FBS. After 24 h of incubation, cells attached on the outside bottom of the upper chambers were fixed with 4% paraformaldehyde and stained with 0.1% crystal violet. For each sample, at least five random areas on the bottom were imaged and counted for the cell numbers. All results were normalized to the average number in the control group.

#### 2.3.5 Flow Cytometry

A375 cells were seeded in 6-well plates at an initial density of 5×10^5^ cells/well and incubated overnight, following which the medium was replaced by fresh medium containing 10 mM of lidocaine as LH or in LNP. Fresh medium containing no drug was used as the control. The cells were incubated with drugs for 4 h and then collected by trypsinization. Using an Annexin V Apoptosis Detection Kit (Thermo Fisher Scientific) and following the manufacture’s introduction, the cells were double-stained by FITC-labeled Annexin V and propidium iodide. Then the stained cells were analyzed by flow cytometry (BD FACSCelesta).

### 2.4 *In vitro* Drug Release

*In vitro* drug release profile of LH and LNP was studied using a dialysis method. Briefly, 1 mL of each formulation was placed in a Spectra/Por Float-A-Lyzer G2 dialysis device (8–10 kDa MWCO, Spectrum Labs, United States) and dialyzed against 30 mL of PBS (pH7.4). At each time point between 0.25 and 48 h, 10 mL of PBS containing released lidocaine were taken out and another 10 mL of fresh PBS was added. The amount of released lidocaine was determined by HPLC method using an Agilent Extend C18 column (4.6 mm × 150 mm, 5 μm), solvent A (10 mM ammonium bicarbonate solution) and B (acetonitrile) with an isocratic gradient ratio of 50:50. The peak of lidocaine was detected at the wavelength of 214 nm. Drug release profiles were constructed by plotting the amount of drug released over time.

### 2.5 Animal Experiments

All animal experiment procedures were approved by the Animal Ethical Committee of West China Hospital, Sichuan University, and conducted in strict accordance with the Guide for the Care and Use of Laboratory Animals by the United States National Institutes of Health. Six-week-old BALB/c-nu nude mice (GemPharmatech Co., Ltd, Chengdu, China) were used to establish tumor models *in vivo*.

#### 2.5.1 Tumor Development Suppression

To compare the efficacy of LH and LNP in suppressing the development of tumor from seeded tumor cells, 1 × 10^6^ A375 cells in 100 µL of serum-free medium were injected subcutaneously into the armpit of the right forelimb of each mouse. The mice were randomly divided into four groups (*n* = 6) for treatment with different formulations including normal saline (NS), 5 mM GQY, 25 mM LH or 25 mM LNP. On day 3 after cell injection, 50 µL of each formulation was injected into the cell lump of each mouse. Starting from day 5 after cell injection, solid tumors were formed and their size was monitored every 2 days by measuring the longest diameter (a) and the shortest diameter (b). The tumor volume was calculated according to the following equation:

$$V=0.5\times a\times b^{2}$$

On day 15 after cell injection, the mice were euthanized and the tumors in each group were collected and weighed.

#### 2.5.2 Tumor Recurrence Inhibition

To compare the efficacy of LH and LNP in preventing tumor recurrence after surgical excision, a tumor excision model was used as described previously (Gao et al., 2019). Briefly, 1 × 10^6^ A375 cells in 100 µL of serum-free medium were injected subcutaneously into the armpit of the right forelimb of each mouse. On day 10 after cell injection, solid tumors with an average size around 200 mm^3^ were formed. For each mouse, an incision was made at the tumor site and 3/4 of the tumor was removed. The mice were randomly divided into four groups (n = 9) for treatment with different formulations including NS, 5 mM GQY, 25 mM LH or 25 mM LNP. To prevent potential drug leaking, each formulation was mixed with 1% hyaluronic acid hydrogel and injected into the tumor site through the incision, after which the incision was sutured carefully. From day 12 to day 40 post-operation, tumor recurrence was monitored by measuring the tumor size every 2 days. Animals were euthanized when tumor volume reached 1,500 mm^3^.

## 3 Results

### 3.1 Formation and Characterization of LNPs

**FIGURE 1:**
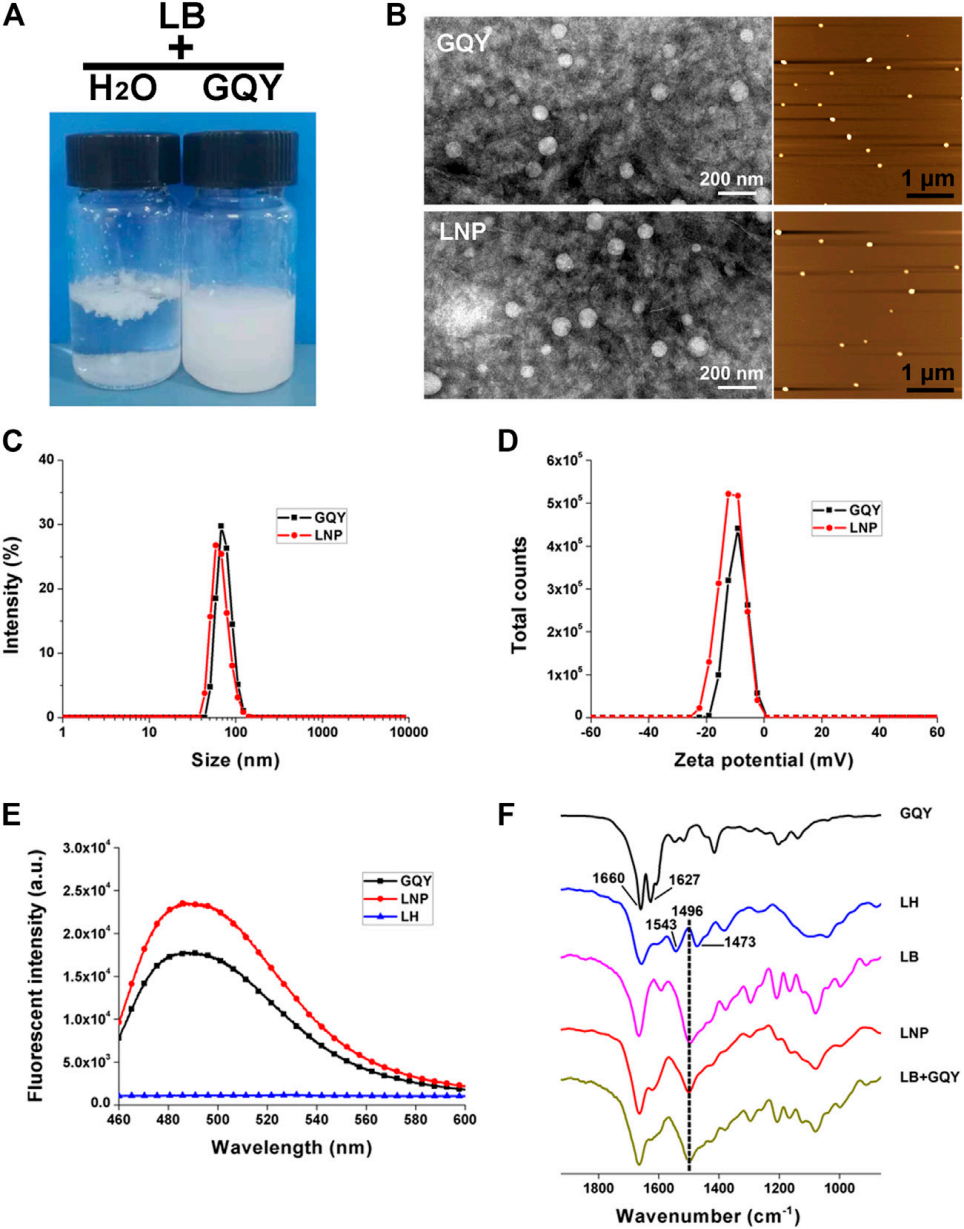
Characterization of LNPs. **(A)** photo pictures of LB dispersed in water (left) or in GQY (right). **(B)** TEM (left) and AFM (right) images of empty GQY nanoparticles and LNPs. **(C)** size distribution of LNPs and empty GQY nanoparticles. **(D)** zeta potential of LNPs and empty GQY nanoparticles. **(E)** ThT-binding fluorescence of GQY, LNPs, and LH. **(F)** FTIR spectra of GQY, LH, LB, LNP, and LB + GQY.

**FIGURE 2:**
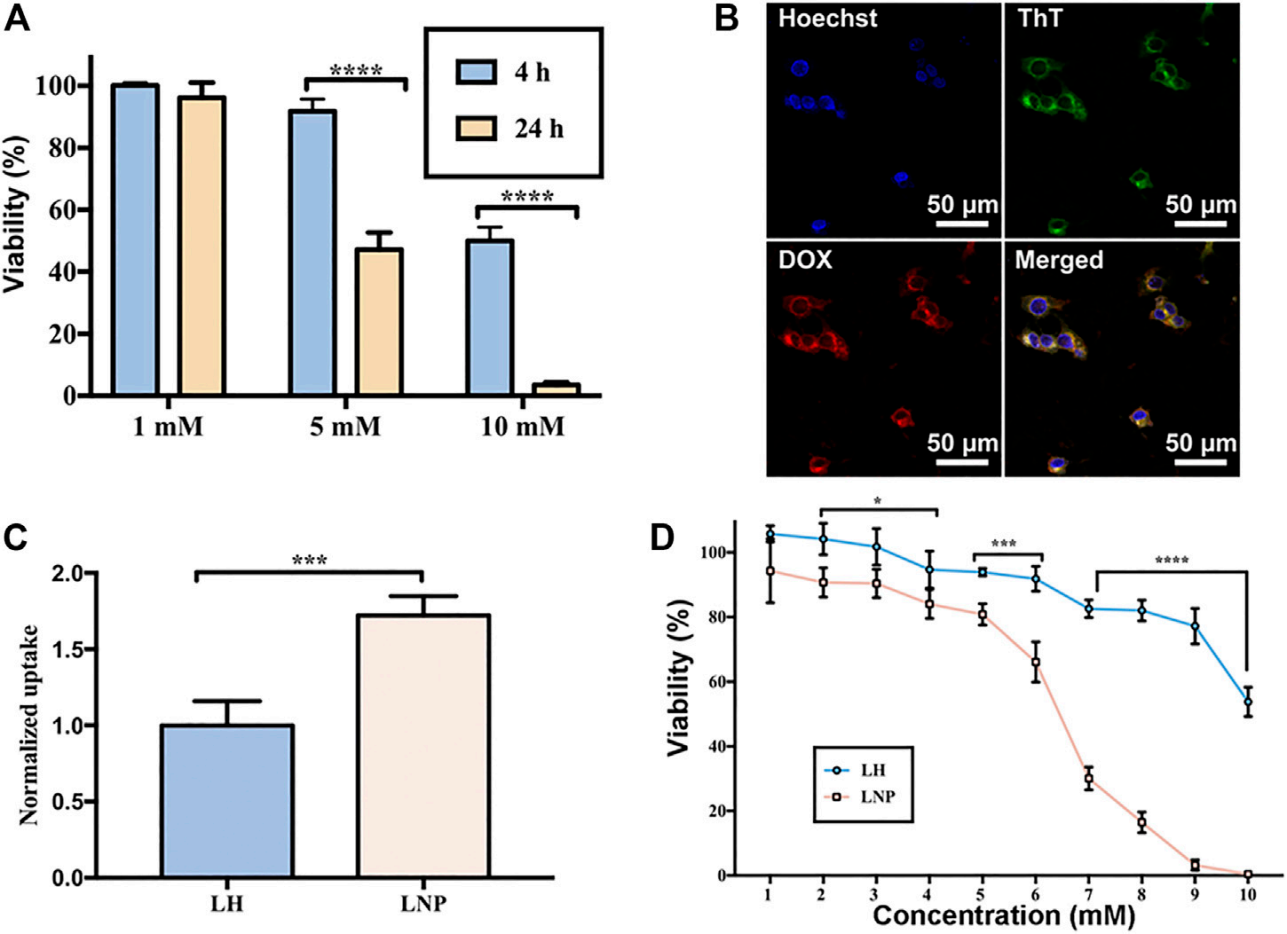
Enhanced cellular uptake and cytotoxicity of LNPs. **(A)** cytotoxicity of LH incubated with A375 cells for 4 or 24 h. **(B)** co-localization of GQY (stained green by ThT) and encapsulated DOX (red) in A375 cells. **(C)** cellular uptake of LH and LNPs. **(D)** cytotoxicity of LH and LNPs with 4-h of incubation. *p < 0.05, ***p < 0.001,****p < 0.0001 by one-way ANOVA with Tamhane’s T2.

As shown in Figure 1A, LB cannot be fully dispersed in water even after vigorous stirring, which confirms its poor water solubility. While in 5 mM GQY solution, LB could be well dispersed and form a stable milky suspension. TEM and AFM images in Figure 1B show that LNPs with a homogenous size smaller than 100 nm were formed, which are morphologically similar to the self-assembling nanoparticles formed by GQY. Furthermore, the particle size distribution shown in Figure 1C indicates that the average size of LNPs in the suspension is similar to that of empty GQY nanoparticles, and zeta potential data in Figure 2D show that the surface of LNPs carries weak negative charge similar to GQY nanoparticles.

The self-assembling behavior of GQY with or without LB was monitored by a ThT-binding fluorescence assay described in our previous study, which could quantify the peptides’ aggregation in a real-time manner (Chen et al., 2018a). As shown in Figure 1E, LNP suspension shows an even higher ThT-binding fluorescence compared with GQY solution at the same peptide concentration, suggesting that the self-assembling behavior of peptide was even strengthened by LB incorporated.

As shown in Figure 1F, the FTIR spectrum of GQY shows two peaks at 1,660 and 1,627 cm^−1^, which can be assigned to amide I band from the peptide backbone. Generally, peptides with very strong aggregation behavior would show only one peak at 1,627 cm^−1^, so that the existence of the peak at 1,660 cm^−1^ suggests that the self-assembly of GQY is not so compact like some other peptides. LH shows two peaks at 1,543 and 1,473 cm^−1^, which can be assigned to the N-H^+^ group of protonated lidocaine, while LB with unprotonated N-H group shows only one peak at 1,496 cm^−1^. Since LNP also shows only one peak at 1,496 cm^−1^, it can be confirmed that in the LNP formulation LB kept its base form and didn’t get protonated. Furthermore, the FTIR spectrum of LNP seems to be a simple summation of the spectra of LB and GQY, and it was also very similar to that of the physical mixture of LB and GQY.

### 3.2 LNPs Enhanced Cellular Uptake and Cytotoxicity

Due to the very short half-life time of LH *in vivo*, cells in solid tumor tissue are expected to expose to LH for only a very short period of time. For this reason, we investigated the antitumor activity of LH under short-term incubation, which can mimic the short-term contact of drug with cells *in vivo*. As shown in Figure 2A, when LH was incubated with A375 cells for 4 h, its cytotoxicity decreased significantly compared with 24-h of incubation.

Using DOX as a fluorescent drug model, it was confirmed that GQY nanoparticles can efficiently deliver hydrophobic drug into the cytosol of cells within 4 h. As shown in Figure 2B, fluorescence of ThT-stained GQY nanoparticles fully overlaps with the fluorescence of DOX, suggesting the co-localization of the carriers and the drug. As shown in Figure 2C, compared with soluble LH, LNPs exhibit a much higher level of cellular uptake. It should be noted that in this cellular uptake experiment, even only after 4 h of incubation, LNPs have already led to the cytolysis of a considerable number of cells as observed by microscope. Since drugs taken by these cells cannot be measured, the actual cellular uptake level in the LNP group is supposed to be even higher. As a result, in a 4-h incubation treatment, LNPs significantly enhanced the cytotoxicity of lidocaine compared with the soluble LH formulation (Figure 2D).

### 3.3 LNPs Inhibited Cell Migration and Invasion

**FIGURE 3:**
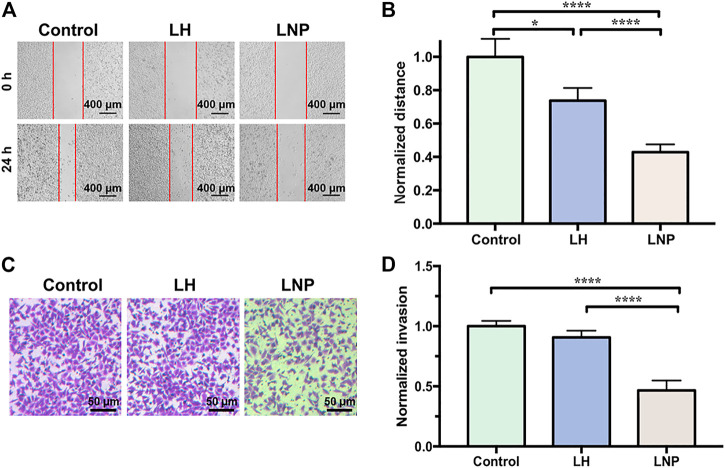
LNPs inhibit migration and invasion of A375 cells. **(A)** representative images of wound healing, with the “wound” area marked by red lines. **(B)** comparison of normalized migration distance between different groups. **(C)** representative images of cells invaded through the membrane of transwell. **(D)** comparison of normalized invasion rate between different groups. *p < 0.05, ****p < 0.0001 by one-way ANOVA with Tamhane’s T2.

Migration and invasion of tumor cells are two important features related to their malignance. As shown in Figure 3, LNPs significantly inhibited the migration and invasion of A375 cells, suggesting a strong antitumor activity. On the contrary, the effect of soluble LH on cell migration and invasion is relatively weak. It should be noted that although the cells were only treated by 4-h of drug incubation, LNPs have already generated a profound effect in inhibiting the cells migration and invasion, which can last till 20 h after drug exposure.

### 3.4 LNPs Induced Apoptosis of A375 Cells

**FIGURE 4:**
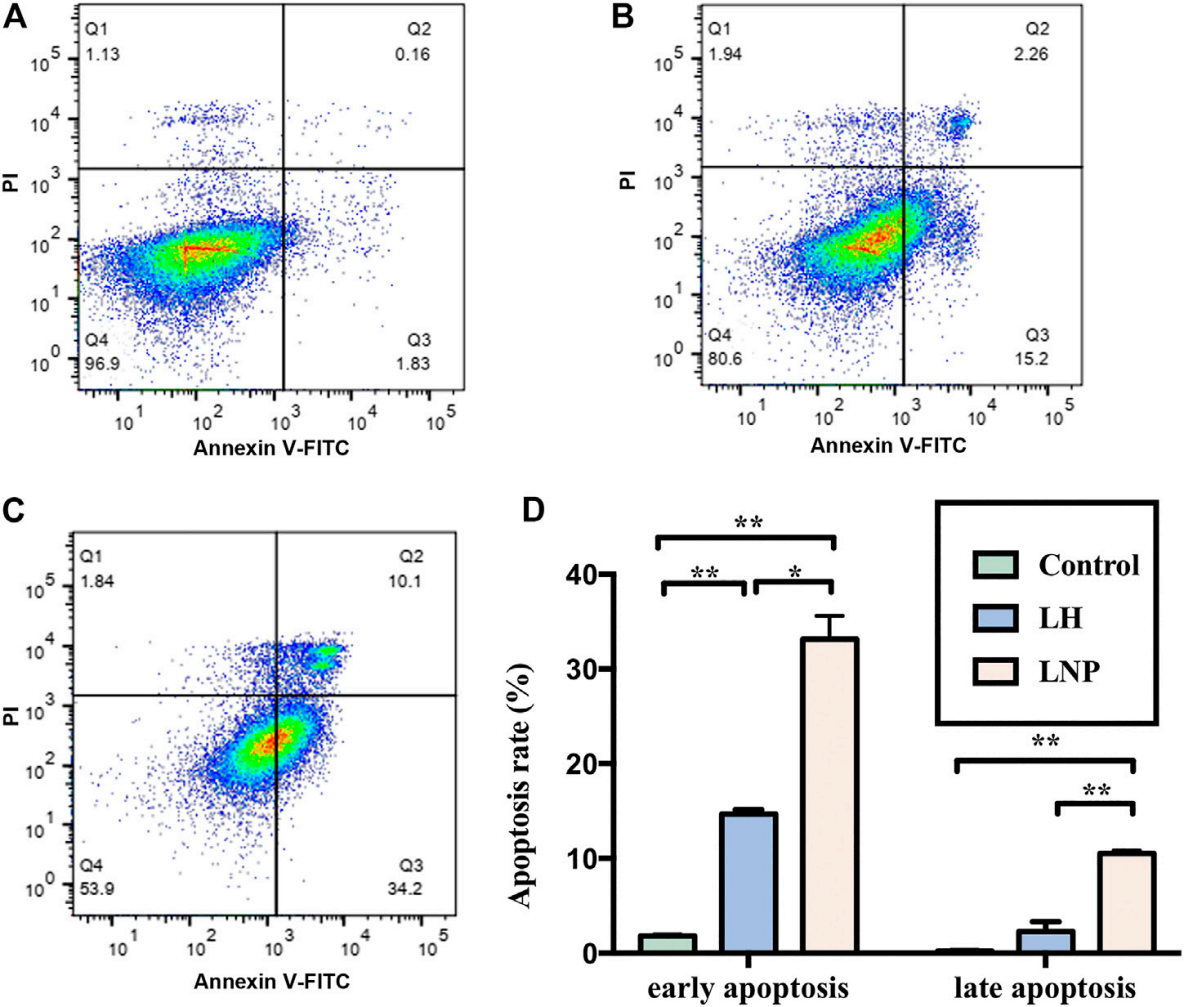
Apoptosis of A375 cells induced by LNPs or LH. Representative flow images were shown for the control group **(A)**, LH group **(B)**, and LNP group **(C)**. Comparison of analyzed apoptosis rate between different groups was shown in **(D)**. *p < 0.05, **p < 0.01 by one-way ANOVA with Tamhane’s T2 post-hoc test.

Although no confirmed mechanism has been established to explain the antitumor activity of lidocaine yet, many studies have suggested that the drug may kill tumor cells by inducing apoptosis (Lu et al., 2011; Wang et al., 2019; Xia et al., 2019). In this study, we incubated A375 cells with LH or LNPs for 4 h to see if they can efficiently induce apoptosis of the cells. As shown in Figure 4, LH only induced apoptosis at a relatively low level after 4 h of incubation, likely due to its inefficient cellular uptake. On the other hand, LNPs significantly enhanced the apoptosis rate of A375 cells, suggesting that LNPs could induce the death of tumor cells through the apoptosis pathway rather quickly.

### 3.5 Slow Drug Release *in vitro*

**FIGURE 5:**
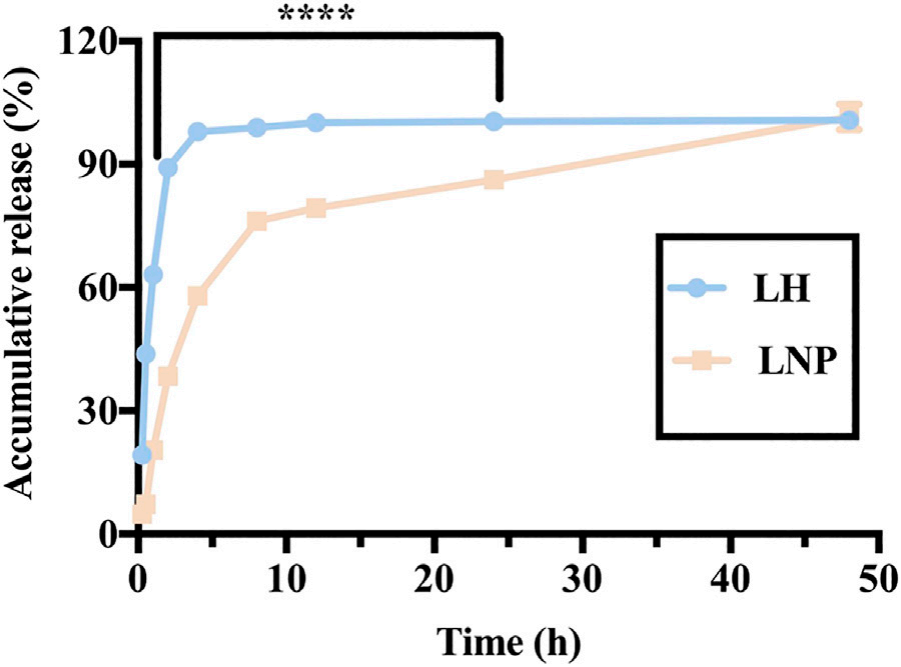
*In vitro* release profile of LH and LNPs. ****p < 0.0001 by one-way ANOVA with Tamhane’s T2.

As shown in Figure 5, in the highly water-soluble LH formulation, 89.13% of the drug was released within 2 h, while only 38.43% of the drug was released from LNPs. Almost no drug was left in the LH formulation only after 4 h, while a considerable amount of drug was retained in the LNPs for up to 24 h. These results demonstrate that LNPs can significantly slow down the release of lidocaine, which is expected to be beneficial for retaining lidocaine longer at the injection site and enhancing its antitumor activity *in vivo*.

### 3.6 LNPs Inhibit Tumor Development and Recurrence *in vivo*

**FIGURE 6:**
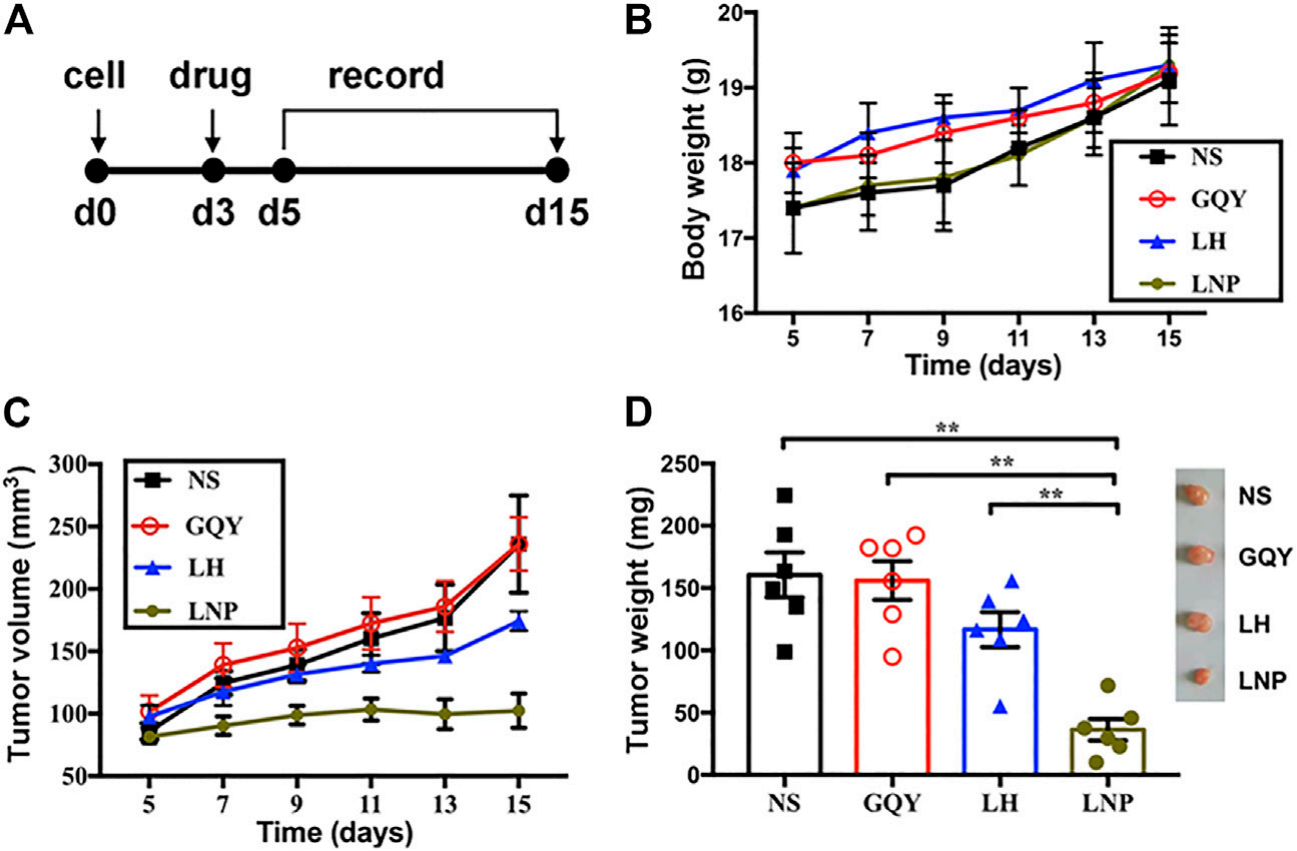
LNPs inhibited tumor development *in vivo*. **(A)** different formulations were injected subcutaneously on day 3 after cell injection and the development of solid tumor was monitored. **(B)** change of body weight of animals during tumor development. **(C)** change of tumor volume measured during tumor development. **(D)** weight comparison and representative pictures (right) of harvested tumors from different groups. **p < 0.01 by one-way ANOVA with Tamhane’s T2 post-hoc test.

To evaluate the antitumor efficacy of LNPs *in vivo*, we investigated their ability to inhibit early tumor development as shown in Figure 6A. First of all, the body weight of animals in all groups steadily increased in a similar trend, suggesting that the formulations used in our study were free of severe side effect (Figure 6B). As shown in Figure 6C a single injection of LNPs significantly inhibited the development of solid A375 tumors in nude mice, while the inhibiting effect of LH is limited and non-significant. As shown in Figure 6D, although tumors could still be formed in mice treated with LNPs, they are significantly smaller than the tumors in other groups. On the contrary, LH formulation with the same lidocaine dosage shows a limited effect in suppressing the development of solid tumors.

**FIGURE 7:**
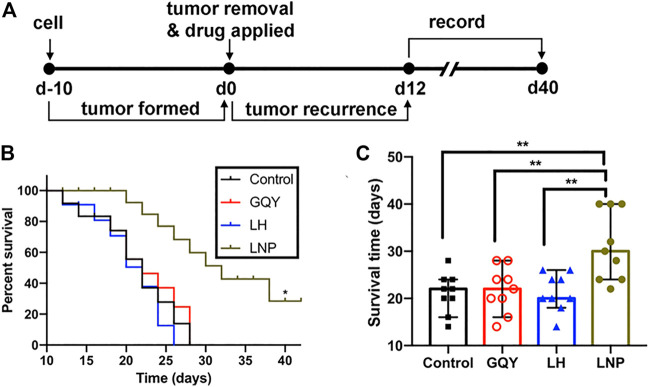
**(A)** preformed tumors were partially excised and different formulations were injected on day 0, and the recurrence of tumors was monitored from day 12. **(B)** survival curve and **(C)** survival time were compared between different groups, determined based on a tumor volume of 1,500 mm^3^. **p < 0.01 by one-way ANOVA with Tamhane’s T2 post-hoc test.

The efficacy of LNPs in inhibiting the recurrence of tumor from residual tumor tissue after surgical excision was also evaluated (Figure 7A). As shown in Figures 7B,C, none of the animals in the NS, GQY and LH groups survived for more than 30 days, with an averaged survival time of less than 22 days. On the contrary, animals in the LNP group survived for at least 22 days, with an average survival time of more than 30 days. Additionally, it should be pointed out that the experiment was terminated on day 40 when the last tumor-bearing mouse was euthanized, so that the longest survival time was set to 40 days. Actually, no tumor recurrence was observed for two mice in the LNP group, which means the animals were completely cured.

## 4 Discussion

**FIGURE 8:**
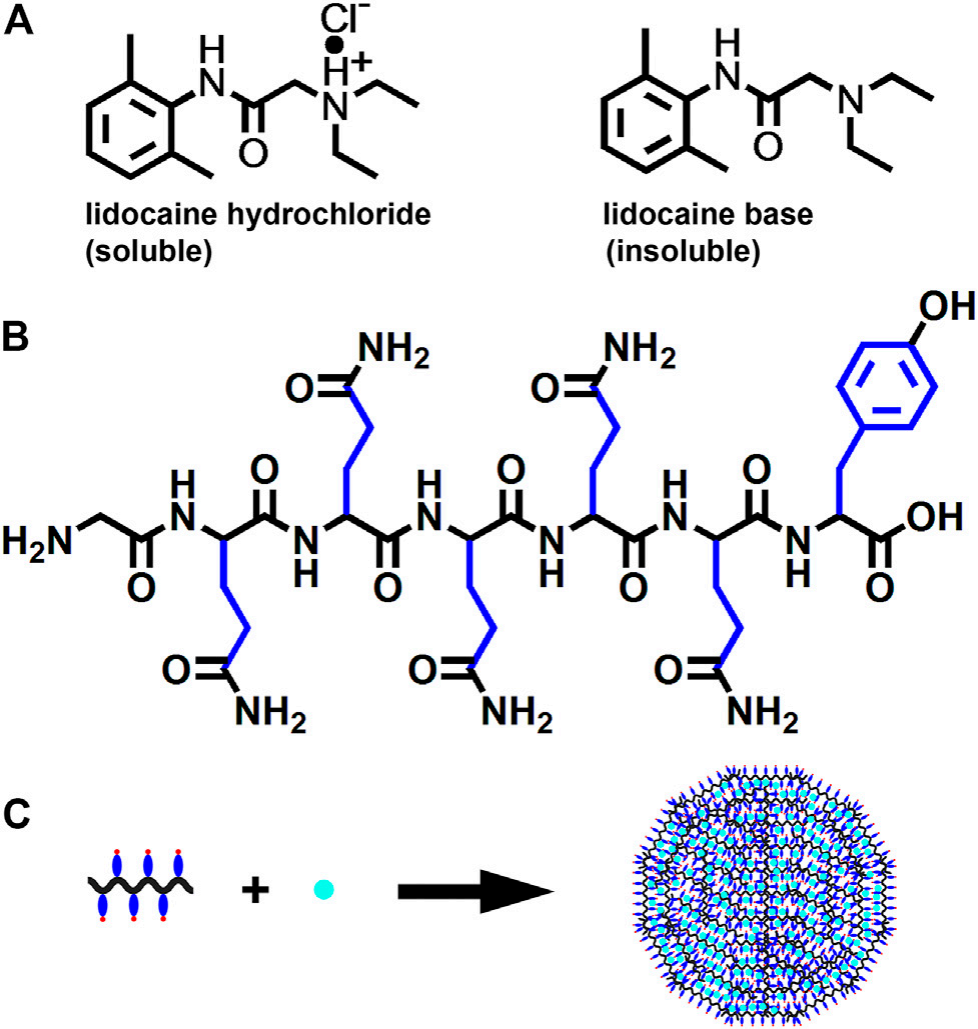
Schematic illustration of LNPs preparation. **(A)** chemical structure of LH and LB. **(B)** chemical structure of GQY peptide with hydrophobic motifs in side-chain shown in blue. **(C)** proposed model for the formation of LNPs.

For many years, LH has been routinely used in clinic practice for its excellent water-solubility, which comes with the positive charge provided by the additional proton (Figure 8A). On the contrary, LB bearing no charge is poorly water soluble, making it a potential candidate for fabricating nanoparticles simply by encapsulating it with carrier materials for hydrophobic compounds. Figure 8B shows the chemical structure of GQY, a designer short peptide containing a glycine residue, five glutamine residues and a tyrosine residue. Although glutamine and tyrosine are conventionally regarded as hydrophilic amino acids, their side-chains contain plenty of hydrophobic motifs, endowing GQY with considerable ability to self-assemble and encapsulate hydrophobic drugs (Chen et al., 2018b). As demonstrated in Figure 8C, with the extensive crosslink among the brush-like hydrophobic side-chains, GQY could self-assemble into nanoparticles with plenty of hydrophobic cavities, in which hydrophobic LB could be embedded.

This encapsulating mechanism is demonstrated by the characterization results shown in Figure 1. The incorporation of LB didn’t change the shape, size and surface charge of GQY nanoparticles drastically, indicating that LB was embedded in the inner hydrophobic cavities of GQY nanoparticles. As shown in Figure 1E, the self-assembling behavior of the peptide was not disturbed by LB. On the contrary, the incorporation of LB even promoted the peptide’s self-assembly, probably by providing hydrophobic cores to induce the aggregation of more GQY molecules at the same peptide concentration.

Additionally, how LB was embedded in the nanoparticles could also be explained by analyzing the FTIR spectra in Figure 1F. Firstly, in the spectrum of GQY, the peak at 1,660 cm^−1^ suggests that the self-assembly of GQY is not very compact, leaving possible hollow cavities inside the nanoparticles for drug loading. Secondly, LNP shows a peak at 1,496 cm^−1^ similar to LB, suggesting that rather than dissolving the drug, GQY is encapsulating the drug without changing its chemical property. Lastly, the spectrum of LNP is very similar to that of the physical mixture of LB and GQY, suggesting that LB is simply embedded in the hollow cavities of GQY nanoparticles without forming extra chemical bonds with the peptide material.

Based on this mechanism, milky suspension with a very high LB concentration could be obtained. The milky suspension shown in Figure 1A could be caused by the self-aggregation of LNPs, since they bear relatively weak charge on their surface. However, it should be mentioned that this milky suspension is still very stable for at least 1 month and could be easily dispersed well by dilution. On the contrary, LB powder cannot be well dispersed in plain water, so that it is not suitable for further cell or animal studies.

In many *in vitro* studies evaluating the antitumor activity of lidocaine or other LAs, the drug was usually incubated with cultured cells for 24 or even 48 h, where they did exhibit excellent cytotoxicity against tumor cells in a concentration-dependent manner. But such a long period of direct drug exposure cannot be possible for an *in vivo* experiment when LH is locally injected. For this reason, in this study we focused on the cytotoxicity of lidocaine formulations under short-term incubation. As shown in Figures 2, 3, LNPs could inhibit the proliferation, migration and invasion of A375 cells more efficiently, which was achieved by enhanced cellular uptake of lidocaine. Furthermore, the results in Figure 4 suggested that LNPs may exert their stronger antitumor activity by inducing apoptosis more efficiently.

Nanoparticles containing antitumor drug can not only kill cultured tumor cells more efficiently by promoting cellular uptake, but also can they release the drug slowly and lead to improved antitumor activity *in vivo*. As shown in Figures 6, 7, LNPs effectively inhibited the development and recurrence of tumors, while the effect of LH formulation was very limited. This is not surprising, since highly soluble LH can be cleared out *in vivo* rather quickly. On the contrary, LNPs as a slow-releasing depot can retain lidocaine at the injection site much longer, leading to a more significant inhibiting effect. Furthermore, it should be noted that these antitumor efficacies were achieved by a single injection of the LNPs formulation. Even better outcome can be expected if the formulation is injected for multiple times.

Although not tested in our current study, it would be interesting to evaluate analgesic effect of the LNPs formulation beside its antitumor activity. Actually, many previous studies have shown that fabricating LA nanoparticles was also an effective strategy to prolong their duration of anesthesia and analgesia effect (Zhan et al., 2018; Li et al., 2019). Since lidocaine as a popular LA has been widely used for perioperative and postoperative analgesia, it would be highly advantageous if its antitumor activity can be exploited and combined with its analgesic effect.

As a biomaterial composed of natural amino acids, GQY didn’t show any effect on the tumor development or health condition of animals, indicating that the peptide is non-toxic and safe. For this reason, the LNPs formulation based on this material is promising for clinical application. Moreover, the GQY peptide can be further ameliorated, for example to bear stronger negative charge to improve the formulation’s dispersity and stability, or to bearing tumor-targeting motifs to inhibit tumor cells more selectively.

Nevertheless, there’re also some limitations for this LNPs formulation due to the nonselective toxicity of lidocaine. Since lidocaine is known to cause severe systemic toxicity if enters the blood stream at high concentration, the LNPs formulation could only be locally applied for better safety. Even though, the concentration of locally injected LNPs should still be controlled to avoid potential reginal toxicity. As a result, the antitumor activity of LNPs within a safe concentration is not strong enough to completely eliminate the development and recurrence of tumor. To solve this problem, a potential direction would be combining LNPs with other antitumor drugs.

There’re two important procedures need to be noted in our study. First, for all cell experiments investigating the effect of different formulations on cultured tumor cells, we incubated the formulations with cells for only 4 h. This is important to keep the *in vitro* condition mimicking the *in vivo* condition as close as possible, since small water-soluble drug injected *in vivo* is usually cleared out very quickly. Second, we evaluated the *in vivo* anti-tumor activity with two different models. In the first model inhibiting the development of solid tumors from planted cells, the number of injected cells can be accurately controlled, providing a normalized starting point for different groups. However, this model cannot mimic the clinical situation when a preformed solid tumor is excised. On the contrary, in the second model we partially removed preformed tumors and use different formulations to inhibit the recurrence of residual tumors. Although in this model it is difficult to normalize the initial size of residual tumors in different animals, it can closely mimic the surgical excision of solid tumor in clinic practice. Combining the two models, the *in vivo* effect of different formulations can be well-evaluated.

On the other hand, there’re also several important works to be carried out beyond our current study. First, the mechanism of how lidocaine and other LAs kill tumor cells need to be clarified, so that we can ameliorate our LNP formulations accordingly for even better antitumor activity. Second, it would be highly advantageous if we can exploit the prolonged analgesic effect of our LNP formulations, which would be an interesting dual-functional formulation for both pain-relief and recurrence inhibiting after tumor excision. And last, our animal experiments only investigated the inhibiting effect on local tumor recurrence. However, metastasis after tumor excision surgery is also an important issue need to be addressed. It would be interesting to investigate if locally injected LNP formulations can also inhibit tumor metastasis and cure the animals more thoroughly.

## Conclusion

In this study, we introduced a simple strategy to fabricate LNP formulation, in which hydrophobic LB was encapsulated by a self-assembling peptide GQY. By enhancing the cellular uptake of active lidocaine, the LNPs showed significantly improved antitumor activity *in vitro* as compared with soluble LH. In animal models, the LNPs formulation also significantly inhibited the development and recurrence of solid tumors. Considering the wide application of lidocaine to relieve the pain caused by cancer-relative surgery, this simple LNPs system can be a promising dual-functional formulation for both pain relief and tumor recurrence suppression. Considering the similarity of other LAs with lidocaine, their nanoparticle formulations can also be exploited using similar peptide material. Following these directions, the application of LAs in postoperative treatment of cancer can be further exploited.

## Data Availability

The raw data supporting the conclusion of this article will be made available by the authors, without undue reservation.

## Abbreviations

AFM, atomic force microscope; DOX, doxorubicin; DLS, dynamic light scattering; FTIR, Fourier transform infrared; GQY, abbreviation for the peptide sequence of GQQQQQY; LAs, local anesthetics; LB, lidocaine base; LH, lidocaine hydrochloride; LNPs, lidocaine nanoparticles; NS, normal saline; OD, optical density; ThT, thioflavin T; TEM, transmission electron microscope.
